# An Essay on Laryngismus Stridulus, or Croup-Like Inspiration of Infants

**Published:** 1836-07-01

**Authors:** 


### Medico-Chirurgical Review,

### No. XLIX.

**[No. 9 of a Decennial Series.]**

APRIL 1, TO JULY 1, 1836.

AN ESSAY ON THE LARYNGISMUS STRIDULUS, OR CROUP-LIKE INSPIRATION OF INFANTS. TO WHICH ARE APPENDED, ILLUSTRATIONS OF THE GENERAL PRINCIPLES OF THE PATHOLOGY OF NERVES, AND OF THE FUNCTIONS AND DISEASES OF THE PAR VAGUM AND ITS PRINCIPAL BRANCHES. By *Hugh Ley*, M.D. &c. &c. Octavo, pp. 480, with Plates. Churchill, London, March, 1836.

[FIRST ARTICLE.]

IF any one should conceive that this is an essay merely on that remarkable affection, which the late Dr. Gooch quaintly termed " CHILD-CROWING," he will be very much and agreeably deceived. Upon this essay, embracing a vast mass of ingenious physiology, important pathology, and valuable facts, Dr. Ley has expended the patient labour of a German, the vivacious acuteness of a Frank, and the masculine reason of a Briton. A perusal of this volume has, indeed, filled us with astonishment how the author, who is yet but a young man—how an active practitioner, who is also a valetudinarian, had been able to collect such a vast granary of illustrations for his subject, from authorities of every age and nation! When it is considered that the work under review embraces an investigation of the PAR VAGUM, together with all its diseased conditions, structural or functional, and those of its branches, especially the recurrents, we need not wonder at the size of the volume, however we may admire the patience and industry of its compiler. This collection of facts and opinions, in the very words of their authors, will, indeed, prove a great treasure to future authors and compilers, as a work of reference, saving them the trouble of original research. It will be a kind of " Burton's Anatomy of Melancholy" to the neurological writer, who will be able to appear very learned without going farther than Dr. Ley's book. At the same time, we freely confess that we do not expect that the whole or half of the work will be generally read, by those who take up the volume to learn the nature and treatment of " LARYNGISMUS STRIDULUS." Readers now-a-days, except when they are preparing to be writers or lecturers, seldom take the trouble of sifting evidence very nicely, or comparing all that has been said on each subject by numerous authors. They are eager to get at the *results* of the writer immediately before them, and leave his various *data* for the literary gourmand. The great majority of his readers, indeed, will wonder why Dr. Ley should take the trouble of collecting so much evidence respecting a disease, which is, comparatively speaking, little frequent, and which, by some writers, for instance Mr. North, is represented as very seldom fatal. These reasons, however, are not valid, for the disease is more common than is supposed, because it is often confounded with other complaints that resemble it—and it is certainly a formidable affection, and not seldom dangerous or fatal. But to proceed at once to our subject.

Dr. L. thinks that, until the beginning of the present century, the disease in question has been described under the vague terms of asthma and struma —" thus associating, as Millar, Molloy, and perhaps Marsh have done, these two affections, which so constantly co-exist in the same individual." Russel and Haygarth are quoted by our author, and it is quite evident that, in 1761, Dr. James Simpson published a thesis in Edinburgh, in which is described the disease in question, under the name—" de Asthmate Infantum Spasmodico," occurring at the commencement of dentition, and consisting of " the most violent paroxysms of asthma, accompanied with a shrill sound— (the clangose sound of Russel)—occasionally destroying life in a single attack—otherwise remitting, and very apt to be reproduced by any violent agitation of the body—continuing its course without cough, or, if any, very slight and dry."

Lieutaud was familiar with the disease, and calls it " catarrhus suffocans." He divides it into two species—one from spasmodic constriction of the glottis—the other from a clogged state of the bronchi.

Dr. J. Millar published a monograph on the subject, and it has gone by the name of " Millar's asthma." Dr. M. practised in a damp and cold locality on the borders of England and Scotland, and remarks that enlargement of the cervical glands was common among children. The following description removes all doubt as to the disease being laryngismus stridulus.

" Children at play were sometimes seized, but it generally came on at night. A child who went to bed in perfect health, waked an hour or two afterwards in a fright, with his face much flushed, or sometimes with a livid colour, incapable of describing what he felt, breathing with much labour and with a convulsive motion of the belly, the returns of inspiration and expiration quickly succeeding each other in that particular sonorous manner, which is often observed in hysteric paroxysms. The child's terror sometimes augmenting the disorder, he clung to the nurse, and, if he was not speedily relieved by coughing, belching, sneezing, vomiting, or purging, the suffocation increased, and he died in the paroxysm."

" But if any of these happened naturally, or was exerted by art, the paroxysm ceased, and the child seemed perfectly well, slept during the remainder of the night, and continued to breathe easily till the following morning, when, if not sooner, he suffered a paroxysm more violent and of longer duration than the former. In very young children, if they were peevish, restless, and cried more than usual, a return of the disease might be expected."—xxv.

In the same year (1755), Rush of Philadelphia published an account of the same disease, addressed to Dr. Millar, and adopting his views. Rutty of Dublin, also, in the same year, notices a similar disease, which raged over an extensive district in Wicklow, Carlow, Kilkenny, &c. carrying off incredible numbers of children. Passing over time and authors, we come to 1781, when Mr. Moss, of Liverpool, describes the disease as prevalent " in the sharp, keen air of the district in which he resided." This account was enlarged and commented on by Dr. Herdman. He comes to the conclusion, that it is neither more nor less than the asthma of adult age. Underwood appears to treat of the disease under the vague term of " inward fits." Wichmann, of Hanover, gives the best account of the complaint which is found in foreign writings. We must pass over Cheyne, Burns, and others, and rest, for a moment, with Hamilton, the present obstetrical professor in Edinburgh. He has described the disease accurately, as may be seen from the following short extract.

" The most formidable symptom (he says), except convulsions, which occurs during dentition remains to be noticed. It is a kind of convulsive stricture of the upper part of the windpipe, producing a peculiar crowing sound, as if from suffocation. This affection is quite momentary, and generally happens on awakening from sleep, or taking drink or food, or on the infant being teazed or irritated. Sometimes the fits are redoubled, but more often they are single. The disease is unaccompanied with fever, or any material derangement of the general health. When cough attends, which is not always the case, it is not hoarse, and the breathing during the intervals is perfectly free. Those circumstances distinguish it from croup, which it resembles in the crowing sound." —xxxix.

Dr. Clarke also has described the disease with great fidelity, and seems to be " the first writer who has noticed its association with a tonic contraction of the fingers, thumb, wrists, feet, and ankles, now well known under the very general designation of 'CARPO-PEDAL CONTRACTION.' " In this our author is probably mistaken. Dr. Kellie, of Leith, has given an account of it in the 12th vol. of the Ed. Journal—and quotes Underwood as having given a vague description of it also. It was the Editor of this Journal who first gave it the name of " CARPO-PEDAL SPASM," in the second volume of the (monthly) Medico-Chirurgical Journal (1817), pp. 448-9. When describing an exquisitely marked case of the disease.*

Of late years, the laryngismus stridulus has become better known through the writings of North, Davies, Pretty, and others. Gooch, who gave it the

---

* *Extract.* " The patient was a delicate female child, nineteen months old, and cutting four teeth at the time. When first seen, the child was seized, three or four times in the hour, with spasmodic affections of the respiratory muscles, consisting of repeated attempts to fill the chest, during which she threw herself back, as in opisthotonos, and appeared on the point of suffocation. The fits lasted ten or twelve minutes, when they declined, leaving the child fretful and irritable. The backs of the hands and insteps were swollen and hard. The thumbs were rigidly contracted, and locked across the palms of the hands. The toes were bent down towards the soles of the feet; and both wrists and ankles were rigidly bent by the flexor muscles, and kept permanently so. The little patient could take no food—was slightly feverish—bowels torpid—stools clayey, slimy, and offensive—eyes heavy and inanimate. For some days, no active treatment was pursued, and the child got worse. Leeches were then applied to the temples—calomel and antimonial powder were given internally—and the warm-bath was exhibited. Under this treatment the child recovered."—*Med.-Chir. Journ,* 1817.

quaint name of "CHILD-CROWING," considered it as probably an affection of the brain, of very fatal tendency. This was the opinion of Mr. Pretty. Dr. Marsh has traced, in the Dublin Hospital Reports (vol. V.), this disease with a masterly hand, connecting its cause with struma, and pointing out its best mode of treatment. So much for the history of laryngismus stridulus. We now come to a description of it.

This formidable malady consists essentially of a constriction of the glottis, impeding the ingress of air into the lungs, and sometimes so completely closing it as to suspend, for a few seconds, the respiration entirely. After vehement, or even convulsive struggles, the child succeeds in getting breath with a shrill sound, like the crowing of a cock, or the inspiration of croup. The attacks are paroxysmal, varying much in duration and intensity. Generally, the intervals are considerable at the beginning. Hours, days, and even weeks may intervene between the fits; but, in the progress of the disease, these paroxysms recur with alarming frequency, there being scarcely a complete interval. The degree of severity increases in proportion. The paroxysms themselves vary much in duration, being sometimes momentary, at others, protracted to a quarter or half an hour. In the early periods, the complaint usually attacks in the night, or after a tranquil sleep; but, when more firmly established, the fits occur at all times of the day or night. The names which the disease has acquired are numerous. Asthma, catarrhus suffocativus, croup, inward fits, spasm of the windpipe, &c. have been its chief denominations. To all these our author makes valid objections; but we need not adduce them here. He thinks the term l. strid. given to it by Dr. Good as the least objectionable. It marks the organ affected, and announces the leading and characteristic symptom. The "child-crowing" of Gooch is, he thinks, equally as good as laryng. stridulus.

"In the violent struggles for the recovery of the breath to which I have alluded, all the muscles supplied by the respiratory system of nerves, are thrown into violent action; the eyes are often involuntarily rolled upwards by the agency of the trochleares; the muscles of the face are expressive of agony, or occasionally convulsed; the head is thrown back by the muscles supplied by the spinal accessory nerve; the serratus magnus is in violent action; the diaphragm and abdominal muscles contract vehemently, and even the extremities are rigid. With all this, however, the face is commonly pallid, and has that lurid tinge denominated cadaverous; and the external veins, turgid with highly-carbonized blood, form black streaks upon the forehead and temples, which continue long after the cessation of the paroxysm." 11.

When the termination is fatal, convulsions not unfrequently precede death. Other symptoms are enumerated by authors; but they are not constant. Among these, may be classed the carpo-pedal spasm already alluded to.

In this place, namely the history, or rather the description of the disease, Dr. Ley suddenly, and rather strangely, plunges at once into the pathology of laryngismus stridulus.

"The cause of the crowing respiration to which I here advert, is either an enlargement of those absorbent glands of the lungs, 'which are constantly found at the root of the lungs, both before and behind the bifurcation of the trachea in the two bronchia, and frequently blend with others which lie upon the arch of the aorta, and not unfrequently between the carotids;' or a similar enlargement of the deep-seated chain of cervical glans, known under the technical appellation of the 'glandulæ concatenatæ.' The former may be enlarged from exposure to cold, from frequent catarrhs, from disease of the lungs, pericardium, or heart, from a strumous taint, and probably from the extension of diseased action from the contiguous cervical glands, which, according to Haller and others, constitute a continuous chain with them; 'quæ cum aspera arteria descendunt, eæ utrumque pariter ejus ramum comitantur, et pulmonis grandia vasa circumstant et anteriores et posteriores.' In the adult, these glands, when morbidly enlarged, may seriously embarrass the respiratory function, and even instantaneously destroy life by suffocation; in children, when similarly enlarged, they may produce the crowing inspiration, preceded or attended by temporary, and even sometimes fatal, asphyxia. To the glands thus seated within the chest, I shall first confine my attention; but numerous instances have fallen within my observation, which prove that similar symptoms may be produced by enlargement of those cervical glands which accompany the trachea in its descent, and the morbid condition of which I shall have occasion afterwards to notice." 19.

For the first hint of this pathology, Dr. L. acknowledges himself indebted to Dr. Merriman. Since then, he has had repeated opportunities of tracing the connexion in question, and of verifying, by dissection, the relation which the symptoms bore to the diseased parts. Some cases are here detailed, which we shall briefly notice.

*Case* 1. A child, while cutting the incisors, became subject to wheezing, which, for a long time, was unaccompanied by any other particular symptom. Expectorants and leeches did not remove this wheezing. Country air had more effect. But the complaint returned with return to town, coming on in fits, chiefly in the night, with disposition to retch. One of them was very severe, and caused great alarm.

"The child was described to have been breathless for a while, pale and cadaverous rather than florid, or even purple, with its trunk and extremities rigid, its hands clenched, the spine bent considerably backwards, the eyes staring and frequently rolled upwards, and the countenance expressive of agony. At last, after an evident and severe struggle, the child recovered, with a shrill sonorous inspiration, like that of the croup or hooping-cough." 23.

An eminent physician considered the head to be in fault, and leeches were applied—but without advantage—rather with injury. Anodynes, with country air, were now employed—and with some benefit—but again the child got worse, and, on returning to town, Dr. Merriman joined in consultation. The child died, and an examination was permitted. Mr. Arnott examined the body.

"All the viscera were healthy. There was no trace of arachnitis, or vascular congestion, or water within the cranium. The abdominal cavity was free from disease, with the exception of a very slight enlargement of some of the mesenteric glands. The lungs were perfectly healthy, spongy, and crepitous. Upon inspecting the bronchial absorbent glands, to which, mentioning my expectations to Mr. Arnott, I directed his peculiar attention, my anticipations were fully realized. The cluster of glands at the root of the lungs, and about the arch of the aorta, were greatly diseased; one of them, as large as a moderate-sized chesnut, being full of the curdled, half-puriform matter, so characteristic of strumous inflammation; another smaller one having also imperfectly suppurated." 25.

We confess that, when we consider that this child died with symptoms, consisting of violent cough, "fever approaching to the character of hectic, and of an unmanageable diarrhœa," we cannot see much ground for the support of the peculiar theory which this volume is designed to confirm.

*Case* 2. A child had attacks of breathlessness, threatening suffocation, ending in crowing inspiration, and accompanied by occasional fits of coughing. He was emaciated, and had had measles and hooping-cough twelve months previously, from which he had never perfectly recovered. The pulse was permanently rapid, with hurried respiration, irregular fever, and circumscribed hectic flush on the cheeks. There was mucous rattle, without expectoration. Dr. Ley apprehended disease of the lungs ; and a consultation was had with an eminent physician, who is not named, but well characterised by "rapidity and general accuracy of discrimination of disease." Our readers cannot be at a loss as to who this physician was. He pronounced the complaint to be hooping-cough. Mineral acids, hemlock, infusion of roses, moderate aperients, and counter-irritants, were unanimously agreed upon. In a fortnight the child died.

"Upon examining his chest, the lungs were found honeycombed throughout their whole extent with vomicæ, varying in size from a pea up to that of a horse-bean or something larger, distinct, circumscribed, and encysted ; but no one of them having, as far as could be traced, any distinguishable communication with the bronchi. There was very considerable enlargement of the bronchial absorbent glands, *to which I was disposed to refer* (as from the symptoms I had expected to find such morbid changes) *the rattle of mucus in the trachea,* the occasional brief suspension of the respiratory functions, the crowing inspiration, the violent spasmodic character of the cough, and its recurrence principally in the night ; such symptoms being highly characteristic of such a pathological condition of the glands within the chest, but very uncommon in phthisis." 27.

We apprehend that few will be disposed to disagree with Dr. Ley in his first diagnosis—though we doubt whether pathologists of the present day will allow that the mucous rattle was the consequence merely of enlargement of the bronchial absorbent glands. It will also be observed, that in neither of these cases was the state of brain examined—nor that of the nerves themselves—the recurrents for instance—whose functions were supposed to be disturbed. We cannot, therefore, conscientiously admit, that these cases have afforded any grounds for the theory of our ingenious author. The following extract opens more clearly the theory of Dr. Ley.

"Examining minutely the preparations in my possession, as well as the enlarged glands in the fatal cases which I have recorded, it was obvious to me that the air-tubes had suffered nothing from pressure ; the trachea retained its ordinary dimensions and form, and the bronchi, where they were cut through as they pierced the glandular mass, had still their regular shape and usual size. The symptoms, therefore, could not be ascribed to mechanical obstruction from compression of the air-tubes. Besides, the effect was produced, not upon the bronchi, where the disease was situated, but upon a mere fraction of the larynx at a distance from the diseased mass, the rima glottidis. This could only be effected by some influence transmitted through the nerves, and the mystery is completely solved when it is borne in mind that these glands are enlarged at the very part where the recurrent nerves turn round to be distributed in their ascent upon the fibrous texture at the back of the trachea, upon the lining membrane of the canal, and ultimately upon the opening muscles of the glottis, to influence the movement of that all-important chink ; and that, in one of the preparations to which I have alluded, the nerve had been carried much out of its natural position, was flattened, and had undergone considerable change of texture from the morbid enlargement of the bronchial glands. From these circumstances it was impossible not to infer that the phenomena were the result of some effect upon these recurrent nerves produced by the enlarged and indurated glands in their vicinity, and, if so, it was by no means irrational to expect that, if a similar impression were produced upon these nerves in their course upwards to their ultimate destination, the muscles upon which the movements of the arytænoid cartilages depend, similar symptoms would be produced. This view of the subject opened a new and important field of investigation, both speculative and practical; and its interesting nature would have amply repaid me for the devotion of much time to the enquiry, even had the practical conclusions to which it leads been unimportant." 30.

A series of cases are now detailed at great length. These we must briefly analyze, because these form the bases of the whole theory, and, without a careful and rigid scrutiny of the data, we cannot expect to come to legitimate conclusions.

*Case* 3. A. W. aged 4 years, rickety and scrofulous, had been for some time affected with cough, coming on in nocturnal paroxysms, with sense of suffocation and sonorous inspiration, resembling crowing. During the day, the child appeared quite well—appetite good, bowels torpid—belly tumid. Dr. Ley directed his attention to the glands of the neck, and found them, from the angle of the jaw to the sternum, and along the clavicle, much enlarged and indurated. Gentle aperients were prescribed, with the mineral acids and tincture of hop. Nutritious diet—warm atmosphere.

" Within a week the cough had sensiby subsided; the child had one fit in the night instead of two, and that had been at once shorter in duration and less severe; the attacks of breathlessness had nearly ceased, the crowing was less marked, and *the glands were evidently diminished in size.* In another week the cough had become slight, and no longer caused any anxiety; and now the glands, which had been before as large as marbles, were reduced to the size of peas. A few days after this the child was sent, for the remainder of the Winter, to Clifton. The mother, who accompanied her, returned at the end of a fortnight, and called, with great thankfulness, to tell me that she had left her child perfectly free from cough or any other affection of its breath, in high spirits, joining other children in their sports, and undergoing as much fatigue as others of the same age; bearing well and with temper the confinement of a day-school, and with the ' lumps in the neck' scarcely to be felt, and not at all to be seen." 33.

All we shall say to this case is—valeat quantum valere debet.

*Case* 4. W. Scott, aged 2 years, was brought to Dr. Ley with strangling cough, chiefly coming on in the night. There was no fever—the limbs were extenuated, belly greatly enlarged, especially above and to the left of the umbilicus—appetite ravenous—digestion imperfect. The whole chain of cervical absorbent glands was found enlarged and indurated. Mild doses of rhubarb, mercury, and soda were prescribed. There was a scabby eruption on the scalp. This was Dec. 4th, 1833. 13th. No return for two or three days of the crowing—cough diminished—eruption disappearing (under proper means)—glands decreasing in size. 26th. No further attacks of breathlessness—the child soon recovered entirely.

*Case* 5. Caroline Rycroft, aged eight months, pale, flabby—father scrofulous—was taken with what was called a fit three weeks previously.

" During the attack the body was never violently agitated with convulsions, but the limbs were rigid and extended, the trunk bent backwards, the hands permanently clenched, after an attempt to grasp the first object within their reach, the countenance at first pallid, then '*of all colours, the face, in fact, like a corpse,*' the veins blueish, and '*showing very much,*' the lips first colourless, and afterwards livid ; and altogether the child 'appeared to be strangling.' From this she at length recovered, with an interrupted crowing inspiration, when, after crying violently for three quarters of an hour, she fell exhausted into a tranquil sleep, which lasted for three hours." 26.

Leeches were applied, and some medicines had been given, since which the child had been better. There had been no return of the fits of great severity ; but she was generally awakened in the night with strugglings for breath and crowing inspirations. She had clenched hands. In the day, this child was comparatively free from complaint. The glandulæ concatenatæ were enlarged. Rhubarb and soda were prescribed. The child recovered without any other remedies.

*Case* 6—*March,* 1834. A physician requested Dr. Ley to take charge of his child, who was subject to fits of crowing inspiration since the commencement of dentition. In other respects the child was in good health. By minute examination, Dr. Ley was able to detect " two pisiform enlargements," by pressing the fingers deep under the sterno-cleido muscle. These glands became more distinct after the lapse of a few weeks. The child had an attack of inflammatory croup during treatment for the other affection. The child recovered.

*Case* 7. This was communicated by Mr. Elwyn, of Poland-street. J. H. aged nine months, became affected, three months previously, with " something peculiar in its breathing." This soon came to " spasmodic crowing," especially in the night.

"The peculiar countenance, the distortion of the limb, and, above all, the visible enlargement of the cervical and even axillary glands, warranted a decided opinion as to the real nature of the complaint. The spasms or fits gradually increased, and more than once I was called in suddenly to witness the death of the child. Upon these occasions there seemed little or no hope of recovery; nevertheless, in time the purple countenance disappeared, the blanched lips resumed their hue, and respiration returned. The child, however, rapidly fell away, and, as it became weaker, the fits returned with less violence but greater frequency, coming on every quarter of an hour." 41.

The child died in three months from the invasion of the symptoms.

" *Post-mortem Examination.* 'The submaxillary, parotid, sublingual, and, indeed, almost all the glands throughout the body, were not only enlarged, but even apparently increased in number. The mesenteric particularly were diseased.

The bronchial glands upon the right side surrounded the recurrent nerve, which was completely imbedded in a cluster of enlarged glands. On the left side there was to be seen but one, and that one not large. It was about the size of a pea, very hard, and seemed to press upon the nerve, squeezing it, as it were, against the trachea. The nerve in its course was accompanied by other glands.'

The larynx was healthy, the lungs gorged from congestion, in some parts almost resembling pulmonary apoplexy. The heart was hypertrophied, the left ventricle being also enlarged." 41.

When we look at the great number of organic lesions discovered in the above dissection, any one of which might account for the death of an infant nine months old, we can hardly bring ourselves to the conclusion, that the enlarged cervical glands played so important a part in the phenomena during life, as Dr. Ley's ingenious theory would imply.

*Case* 8—*Dec.* 19*th*, 1833. W. C. aged 15 months, had been suckled by the mother. In the preceding March, the child had violent convulsions, and was largely bled from the jugular vein. Calomel, in smart doses, had also been given. From that period, there was a depression of the globe of each eye, the motions of which, however, were not otherwise altered. The child was now pale, flabby, extenuated—belly tumid—bowels torpid—stools offensive—no symptom of cerebral disease. It presents the carpo-pedal spasms.

"This child often awakes suddenly in the night, struggling for breath, with its hands clenched, its limbs rigidly extended, the spine bent, as in opisthotonos, the head thrown backward, the eyes staring, the face pallid and death-like, the lips colourless. The nurse is in constant fear that it may strangle, and therefore 'pats it upon the back, and shakes it violently to bring about its recovery.' At length, after vehement struggles, the breath is partially recovered with a sonorous inspiration, and the sound of '*phlegm in the throat*' is heard. Soon after this a fit of screaming ends the attack, and the child falls asleep." 42.

The neck was found studded with enlarged and indurated glands, like marbles or bullets. The gums were freely lanced, and the hyd. cum cretâ, with small doses of ipecacuan, rhubarb, and soda were given. The child was sometimes better, sometimes worse. On the 30th Dec. however, it had a "dreadful night," and the dyspnœa was so great, that the parents thought it would die. There were *now* symptoms of cerebral congestion, and leeches were applied to the head, with more active aperients, tepid bath, &c. The little patient improved gradually but greatly, and Dr. Ley left off attendance. Some time after this he was sent for in the night; but, being out of town, another practitioner was called in. The child recovered from that attack, but, a few days afterwards, it was seized suddenly in the night, and died before the practitioner could get to the house. Leave was obtained to examine the body. There were some thickenings of portions of the arachnoid, and slight serous infiltration between the membranes, but no water in the ventricles. The brain itself appeared more pallid than common. "Several glands were detected over the course of the recurrent on each side; and, upon the left side, a little above the clavicle, that nerve was completely imbedded in a surrounding glandular mass. The bronchial glands were not sensibly enlarged. The mesenteric glands were very considerably enlarged and indurated."

"Many other cases of the dependence of this disease upon glandular enlargement, some of which I shall have occasion to mention hereafter for other purposes, have fallen within my observation, and I can assert with perfect confidence, that in considerably above twenty successive instances, with one exception only, I could trace the enlarged glands either from the commencement, or in the progress of the complaint; they have been either distinguished during life or discovered after death."

"I may now, therefore, I trust without arrogance, assume that enough has been said to establish the proposition, that at least a large numerical majority of instances of the crowing inspiration of infants are produced by the enlargement of glands, situated in the course and influencing the functions of the recurrent nerves, and even sometimes, probably, the par vagum. Some may perhaps be disposed to refer the disturbed state of the respiratory function in these cases to mechanical obstruction by direct pressure of the air-tube itself producing a diminution of its area. But, without entering minutely into this question now, I may content myself with alleging, in anticipation of such an objection, first, that there is no evidence of such a diminution of the calibre of the trachea; secondly, that infinitely larger tumours, as the goitre, situated directly over the trachea, produce no such effects; thirdly, that this canal will bear a degree of pressure from a hard and circumscribed tumour, which may produce ulceration of its mucous membrane and ecchymosis, without the occurrence of similar symptoms; fourthly, that the symptoms of this disease are not those of structural change producing mechanical obstruction of the trachea, as must be clear to all who are familiar with the phenomena arising from inflammatory deposits in that tube; and, lastly, that the seat of the disordered function does not correspond with the seat of the actual disease, which are, in fact, out of the reach of each other:—and these considerations lead me irresistibly to the inference, that it can only be by nervous communication between the disease and the muscles of the glottis, that the contraction of that chink, upon which the breathlessness and crowing inspiration depend, can be produced." 49.

Thus terminates the HISTORY of laryngismus stridulus. The second chapter embraces the—

### CAUSES OF THE DISEASE.

The *predisposing* causes are *age*, since it is almost universally confined to infancy and childhood—constitution (it runs much in families)—climate and season (dampness and cold)—scrofula—bad diet. The *exciting* causes are placed by our author to the account of dentition—inflamed scalp—cerebral congestion—bronchitis—pulmonary disease—pericarditis.

### CAUSES OF THE PAROXYSM.

Straining of the body—exercise—fretting and crying—cough—abdominal distention—suddenly awaking from sleep—fright—efforts to swallow—sudden application of cold. The investigation of these causes, predisposing and exciting, occupies 54 pages of closely-printed matter. We conscientiously believe that the author might have compressed all that is valuable in those 54 pages into half-a-dozen.

The following comprises the pith of the section on etiology.

"It results from this examination, that the predisposing causes are such as have a tendency to produce,—and do actually produce,—glandular enlargement, or to call into action a scrofulous tendency; that the exciting causes are such, as are peculiarly apt to occasion enlargement of the cervical or thoracic absorbent glands; and that the causes of the paroxysm are such as, by natural association without morbid influence, involuntarily close the glottis; that the breathlessness, which commonly precedes the sonorous inspiration, cannot, therefore, be from this closing of the rima glottidis, which, in such cases, is perfectly normal, but must arise from defective power in those agents, whose office it is to open that chink; and, lastly, that the crowing inspiration, which is Nature's imperfect cure of the temporary suspension of breathing, arises from the chink being only partially open for the admission of air, and remaining so until some explosive expiration, such as screaming, crying, coughing, or belching, shall mechanically burst open the flood-gates, and perfect the recovery from the paroxysm." 103.

### Chap. III.—Pathology.

This chapter occupies half a hundred pages, though the subject would seem to us to have been considerably developed in the introduction and in the first chapter, entitled the History of the Disease. We suspect that few of our readers are now to learn what Dr. Ley looks upon as the pathology of laryngismus stridulus. In combating the opinions of Rush, as to the spasmodic nature of the disease in question, our author observes that—

"It thus appears, that by three several morbid conditions, the pasage of air into and through the larynx may be impeded or interrupted, 1st, by inflammatory deposit producing mechanical obstruction, and interfering with the movements of the arytænoid cartilages, as in croup; 2dly, by spasm of the closing muscles of the glottis, as in the earlier stages of croup, perhaps in hooping-cough; and, lastly, by an enfeebled or paralytic state of the opening muscles of that chink:—and it is more than probable that 'there is often a mistake made, by considering the contraction of one set of muscles produced by torpor or paralysis of the antagonists for spasm.'" 107.

The connexion of this disease with disturbance of the cerebral circulation, Dr. Ley admits, does sometimes, though very rarely exist. He believes, however, that such disturbance is generally owing to the frequent interruptions to the breathing which take place in the paroxysms—and, therefore, that it is a consequence rather than a cause of the disease. The following principles will be admitted without dispute, as resulting from a comparison of a large series of facts.

"1st, That morbid impressions upon nerves produce their effects upon the remote distribution of their filaments; 2ndly, that all the branches of a common trunk have a similar disorder of function from the same morbid impression; 3dly, that the diseased conditions of nerves resolve themselves into those of excitement, upon the one hand, and those of defective energy upon the other; 4thly, that excitement, of which the most frequent causes are sudden mechanical impulse, vascular congestion or inflammation, structural disease, and sometimes simply functional disorder, is characterised by what modern continental writers delight to call an 'exaltation of function,'—a morbid or anormal excess of nervous energy; and, 5thly, that diminished energy which is commonly the consequence of some extraneous pressure, or of a solution of continuity, the result of accidents, of ulceration, or of wasting, is characterised by an enfeebled condition of the parts upon which the affected nerve is distributed, in all its various degrees, from simple torpor or 'benumbment,' up to the most complete paralysis." 120.

The glands affected in this disease, Dr. L. remarks, are all seated below, and frequently beyond the reach of, the superior laryngeal nerves, which nerves, therefore, preserve, unimpaired, their ordinary functions.

'Supplying, as they do, the closing, to the exclusion of the opening, muscles of the glottis, together with the lining membrane of the upper edge and surface of that chink, the rima glottidis continues to be closed in those associated actions, which are always accompanied with a temporary approximation of its sides.

Such are all-powerful exertions of the body, swallowing, crying, sobbing, violent cough, and even fright, as well as the accidental admission of any irritating substance within the chink, which, from its extreme sensitiveness to external impressions, is commonly denominated the '*sensible glottis.*'

It must, consequently, be either upon the par vagum, below the origin of the superior laryngeal nerves, or upon its recurrent branches, that the morbid influence of these glands must be exerted. If the cervical glands be exclusively in fault, I believe that the par vagum will be rarely implicated. These nerves are so deep-seated, are so close to, and even frequently under, the edge of the carotid artery, between which and the jugular vein they are commonly situated, that any agent which could there mechanically influence the nerves, could not fail, also, materially to interfere with the circulation in those vessels, of which, however, there is commonly no decisive, if any evidence. But these glands are imbedded in the same cellular matrix with that which surrounds, and to a certain extent protects, the recurrent nerves; and they constitute, when in a state of morbid induration and enlargement, hard nodules in such direct contiguity and contact with the latter nerves, that these can scarcely escape their injurious effects.

If those general propositions connected with the pathology of nerves, which I have ventured to announce, are well founded, there can be little difficulty (the seat of the morbid impression being ascertained) in understanding where the symptoms will manifest themselves, and what will be the nature of those symptoms. If it be true, that such morbid impressions upon the trunks of nerves produce no effect above, and little, if any, at the precise point which is the seat of injury or disease, but principally, if not entirely, at the remote distribution of their filaments, the functional disturbance of the recurrent, consequent upon disease or injury, will betray itself in the transverse muscular bands connecting the extremities of the rings of the trachea, in the sensibility of the mucous lining of that canal, and in the movements of the glottis; and the morbid effects will be restricted to the trachea and larynx, for upon these parts alone is this nerve distributed. But if the par vagum be also affected either above or below the part which gives off the recurrent, the heart may suffer, though, according to experiment and observation, only temporarily and inconsiderably; the lungs, and especially the air-cells, may be seriously affected; and the stomach, particularly its larger extremity, will not escape.

If, again, it be true that all the branches proceeding from a common trunk have a similar disturbance of function from the same injurious impression, then, if one part which derives its energy from a given source be excited, all the other parts deriving their supply from the same common trunk will be similarly affected; so, if defective energy manifest itself in one portion of the distribution of the recurrent nerve, it may be expected that the rest of its branches will be affected with similar torpor; and since, in the laryngismus stridulus, there is no evidence of the operation or existence of any of those causes which produce excitement of a nerve; since the glands, with the enlarged and indurated states of which the disease has a common and all but invariable connexion from their texture, form, and situation, are infinitely more likely to produce direct compression than sudden impulse, neurilematous congestion, inflammation, or structural change of the medullary matter of the nerves themselves, it appears, at the least, extremely probable, that this disease is more nearly allied to partial paralysis from pressure, than to convulsive movement from morbid excess of energy or of action in the nerves affected." 124.

Dr. Ley admits that "this may appear a startling proposition," especially to those who have habitually looked upon the disease in question as one of spasm. Dr. L. thought for a long time, *more majorum*, that spasm resulted from excess of nervous energy. But reiterated observation induced him to think otherwise. The symptoms, he remarks, if minutely examined, are not those of excitement, either of the par vagum or recurrents. On the contrary, they bear a striking resemblance to those resulting from division of those nerves, in physiological experiments.

"This condition of the nerves, whilst it explains satisfactorily all the phenomena, derives essential confirmation from the structural changes observable in the nerves themselves. They will be frequently found flattened, so as to resemble tape, and so thin, as partially to transmit light: upon other occasions, however, although they may preserve their rounded form, they become atrophied or withered. These changes I have seen in many instances, and they are strikingly exemplified in the preparation from which the annexed engravings are taken. In no one instance have I been able to recognise the slightest appearance of inflammation, which always, where the *atrophie* is the result of such disease, is found upon the borders of such withered portion; there has been no trace of the bright redness, swelling, separation of the filaments from each other, close network of vessels, increased vascularity of the interstitial cellular tissue of the nervous cords, which, according to M. Gendrin, are the anatomical characters of acute neuritis; nor the serous infiltration, the thickening, and induration of chronic inflammation; nor the ramollissement of gangrenous inflammation; nor the deposit of pus amongst the filaments, the consequence of suppurative inflammation, noticed by M. Martinet; and, in the absence of all evidence of the existence of such conditions, even in any part of the course of these nerves, we are not entitled to assume them. The inference, therefore, from all this appears to me irresistible, that it is in consequence of the compression and consequent interruption to the function of these nerves that *atrophie* occurs; and this affords additional evidence, where it is found, that the cause of the symptoms throughout has been paralysis from simple pressure.

Such are the grounds upon which I rest the doctrine of the dependence of this very peculiar complaint upon paralysis of the muscles supplied by the recurrent nerves, rather than upon convulsive movement of those, which derive their energy from the superior laryngeal branches of the par vagum." 134.

Our author next proceeds to notice and answer many objections that have been offered to his doctrines, as propounded in our contemporary, the MEDICAL GAZETTE. We confess that, to our apprehension, he has not been very successful on these points. One reason of this failure appears to be, a less amount of ingenuity than the author exhibits upon other occasions. Thus, to the objection "*that, if these glands acted upon the principle of compression, the pressure being permanent, the symptoms should be so too,*" Dr. Ley has replied in arguments of a rather feeble character. As the objection of permanency of cause and periodicity of effect is the greatest stumbling-block which Dr. Ley meets with, we shall allow him to speak for himself—and we will also help him a little on the occasion.

"Some, whose opinions I respect, see in the paroxysmal character of this disease, an argument against its dependence upon enlargement of glands, which, being a permanent cause, should according to them produce a permanent effect. This, however, applies at least with equal force to the other assigned causes, and especially to vascular turgescence or excitement within the cranium. But, although it may be true that the enlarged glands constitute a permanent disease, it by no means follows that pressure is constantly exerted,—at least to an extent which is sufficient to paralyse the nerve. In some positions, and probably in a relaxed state of the muscles of the neck, there may be no morbid influence exerted. A gland, which, when the neck is straight and quiescent, is not directly over the nerve, may be brought in contact with its surface upon a sudden change of posture; and the muscles, then in vigorous action, may at the same time so bind down or push inwards the tumour, as to produce a morbid effect; hence, in some instances, we hear of the fit being produced by a sudden twisting of the neck. Or the gland, although constantly over the nerve, may require additional compression to produce the effect; hence a fit of crying, or of coughing, or even of hiccough from flatulency, will not unfrequently prove the occasional cause of a fit, because, in these states, the muscles of the throat are notoriously in violent action. Again, the causes may vary in kind and intensity, and the size, permanence, and hardness of the tumours will vary accordingly. A bubo from simple irritation, as from chafing, will rapidly subside upon soothing the local cause; and so in this disease, within a few hours from irritation of the gums or membrane covering a tooth, the glands swell, and the paroxysms occur in rapid succession; but, upon lancing the gums freely, the excitement is subdued, the glands diminish, and the fits often first become less frequent and shorter in duration, and then entirely disappear.

But the pressure, and possibly even the paralysis, may be continuous, and yet the manifestation of this state be occasional only. This, it is now well known, is often the case in paralysis arising from local pressure upon the respiratory nerve of the face, as has been satisfactorily shewn in the papers of the late Mr. Shaw upon this subject, and in the observations of Sir Charles Bell. In these cases, the defective power is often not observed until speaking, mental emotion, or difficulty of breathing, call these muscles into vigorous action; then, one set of muscles acting to the exclusion of their antagonists, the face is distorted. It is probably the same with the glottis. Although physiologists speak in general terms of the opening and shutting of this important chink in ordinary respiration, it must be obvious that in neither of the alternate actions of the respiratory function can it be completely closed. In inspiration it must admit the entrance inwards, in expiration, it must equally allow the escape outwards of the air; in both, therefore, it must be pervious; and as to the slight period which intervenes between the two, the labours of the physiologist have taught us nothing upon a point, which, after all, perhaps, is scarcely worthy of inquiry. We know, however, that the medium state of this elastic chink, when uninfluenced by muscular agency, is that of permeability to the ingress and egress of air. It is so in the still-born fœtus; it is so when all nervous energy is annihilated by the division of the four laryngeal nerves, as was ascertained by an experiment of Majendie. In the tranquil slumbers of the infant, and even in an adult who is in perfect health, no expansion of the nostrils, no raising of the shoulders is observable during sleep; and it is probable that the same thing obtains with respect to the glottis. We are unconscious of any movement of this part in ordinary respiration; we know that it is always open unless closed for some special purpose, as to prevent the intrusion of an extraneous substance into the windpipe; it even opens, after it it has been closed, in that short expiratory action, which, in the ordinary state of things, immediately succeeds the effort to swallow, and which it requires a positive and almost painful exertion to interrupt. It is upon the whole, therefore, more than probable, that in ordinary and easy respiration, the muscles of the glottis are little, if at all, exerted. But when the breathing is embarrassed from any cause, as in the experiment of Le Gallois, when he separated the larynx from its attachment to the os hyoides, that he might see the movements of the glottis, or after the half-accomplished purpose of the suicide, when the movements of the glottis were exposed to the view of Sir Charles Bell and others; then an obvious and even considerable opening and shutting of the glottis may be seen. And, as paralysis of the muscles supplied by the respiratory nerves of the face and neck, in some cases only manifests itself when the muscles of one side, in a healthy condition, act unopposed by their antagonists, so, in the glottis, the effects of the enfeebled and even paralysed state of its opening muscles is only observed in those more vigorous efforts, which are made when the respiration is hurried or impeded—as in fright, in sudden waking from sleep in consequence of some strong external impression, in screaming or crying, in coughing, or when the descent of the diaphragm is prevented by distention of the alimentary canal, whether from the accumulation of indigested or feculent matters, or simply from flatulency, the result of indigestion. All violent exertions of the body will, by the closure of the glottis which such exertions imply, occasion a paroxysm. So, according to Millar, 'children at play were sometimes seized ;' according to Clarke, 'the attacks are often brought on by straining, by exercise,' &c. ; and these facts curiously correspond with the results of the experiments of Le Gallois, who found after the division of the recurrents in dogs of the age of three months or more, that the embarrassment of respiration was not sufficient to destroy them ; 'cats, however, were more inconvenienced, *and if agitated or forced to walk, often fell down as if suffocated.*' In all these cases of vigorous and forcible muscular exertion, the closing muscles of the glottis, acting without antagonists, cause such an approximation of the sides of the glottis, as, existing in one degree, may occasion a slight wheezing ; in a second, and greater, may produce the crowing inspiration from the rush of air through the constricted aperture ; and in a third, and still greater degree, may hermetically seal the larynx against the admission of air, occasioning asphyxia. This latter state is generally only temporary, and is commonly followed by the sonorous inspiration, which is Nature's remedy rather than the essential disease ; and this natural cure is brought about either by the gradual cessation of action of the closing muscles, in conformity with that universal law of muscular motion, that after contraction the fibres will relax of themselves, or from some temporary cause of pressure, which had influenced the nerves by forcing the glands inwards, being diminished or removed. Then, nervous energy being restored to the recurrent, the parts which it supplies recover their tone and power of action, and again open the chink for the purpose of respiration. More, perhaps, is to be ascribed to the restoration of the power of contraction in the opening, than to the cessation of action in the closing muscles, as it is found that, if the inability to inspire continues uninterruptedly for the space of about two minutes, the child then falls a victim to the disease; in the language of Capuron, 'l'enfant a été réellement suffoqué.' Had the cause of the closing of the chink in these cases been spasm of the closing, rather than paralysis of the opening muscles, it might have been expected that, in the state of relaxation of all the muscular textures preceding death, such spasm would have relaxed, and the breathing have been re-established. This happens in the most violent convulsive maladies with which we are acquainted. In fatal cases of epilepsy and puerperal convulsions in adults, and of true convulsions from cerebral excitement in infants, it is almost invariably found that it is rather in the comatose state which succeeds the attack, than in the convulsions themselves, that death commonly occurs; and even in hydrophobia, a cessation of the paroxysms generally occurs for some hours previously to the death of the patient. A very slight degree of power, applied in the proper direction, would be sufficient to open the glottis in these attacks of laryngismus ; but that force is lost because the muscles, which should exert it, are paralysed. In the similar condition produced by complete division of the recurrents, air could not be drawn from above downwards by the agency of a syringe, the resistance being like that produced by putting a finger upon its extremity ; but in the opposite direction, from the trachea upwards, air could be injected by the employment of very inconsiderable pressure upon the piston. So a very moderate action of the opening muscles, when they have regained their influence, may suffice to render the glottis again pervious ; or it may be that in screaming, with which the fit often terminates, and in coughing, which is not uncommon, the expiratory effort acting, like the syringe, from the lungs upwards, may produce mechanically the same effect, and open the chink." 149.

But why did not Dr. Ley ask those who object to *permanency of cause*, as inconsistent with *periodicity of effect*, to explain numerous examples of this same inconsistency, which every day occurs to our observation? Is not the cause of an intermittent fever, in Walcheren, or Batavia, or Lincolnshire, present every day, though the effect is perceptible every other day? If it be replied—"Oh! this is not an *organic* cause"—then we meet our objector on his own ground. The man who contracted ague in a malarious district at last leaves that locality, and removes to a healthy situation. But he carries with him enlarged spleen or liver—and this enlargement will produce as complete an ague as ever was seen in the world. Here the cause is organic and permanent, though the effects are periodical. Where, we would ask, is there a disease, whatever be its cause, that does not occasionally assume a periodical character? Fever, the most general and continued of all, has its diurnal exacerbations and remissions—nay, its complete remissions. Asthma, which is now universally allowed to be most commonly dependent on organic disease of heart or lungs, has its periods.

Tic douloureux, however organic and fixed may be its cause, has its periodical attacks, however irregular they may be—and so has the whole class of neuralgiæ. Look at insanity, which every one now allows to be a bodily disease—has IT not its paroxysms and lucid intervals? Even MONOMANIA has been repeatedly proved to be periodical, though the causes must be permanent. A man has a delusion every other day, week, or month. Is it rational to suppose that the cause of this takes place at such epochs only? It is quite ridiculous to entertain any such supposition. In fine, every malady to which the human frame is subject has, occasionally, a tendency to periodicity, whether the cause be of an organic nature, demonstrated by the scalpel, or occult, and incognizable by the senses. And, so far, we regard this paramount objection to Dr. Ley's doctrine as perfectly futile, and rebutted by every day's experience. Still, we fear that considerable doubts will be entertained by a large majority of the profession, respecting the ingenious pathology broached and sustained by our author. We confess, indeed, that we ourselves are not quite converts to the doctrine. There is something about it, or in it, which engenders scepticism in our minds; and we need not say that we are unable to *compel* our belief or faith on any subject, whether in physic, politics, or religion. The frequent connexion of carpopedal spasm with the crowing inspiration, is not well accounted for by the present theory.

The fourth and fifth chapters, on diagnosis and prognosis, occupying between thirty and forty pages, we must pass over without notice. Dr. Ley has done himself and his subject great injury, by unconscionable prolixity. Laryngismus stridulus, in the hands of Dr. Ley, has occupied more space than did the "Practice of Physic" in the hands of the immortal Cullen! Whatever authors may avow and protest, we strongly suspect that one principal object of writing is—*to be read.* Now we venture to predict, that the whole of this volume, valuable and erudite as it is, will never be read by any one individual in this world. Those who are engaged in actual practice will not be able to afford the *time*—those who are waiting for avocation, will not be inclined to dedicate the *labour* for such perusal. Est modus in rebus, is a maxim which authors, and especially medical authors, should never forget.

### Chap. VI.—Treatment.

This department, occupying nearly one hundred pages, will probably be that most carefully perused—though, even here, the besetting sin of our highly-gifted author—prolixity—is not a little conspicuous. And not prolixity alone—but repetition.

There can be no doubt that, in a disease whose pathology was so little understood, the remedies have been rather enumerated, than arranged or explained. They have been suggested by some loose analogy, or sanctioned by downright empiricism. If the experience of the profession shall confirm the connexion of the complaint in question with enlargement of the cervical or absorbent glands, then we shall have a clue, or rather a key, to the therapeutic part of the business.

"In the treatment, therefore, of this complaint, it should be a primary object to ascertain if these glands are enlarged, and tracing, if possible, the producing cause of such enlargement, to adapt our remedial measures to that cause. Nor must we, simply from difficulty in detecting its existence during life, conclude that there is in reality no such pathological condition. Tumid glands may, as has been already shewn, escape detection, even when seated in the neck; whilst, if situated within the thorax, they are always beyond the reach of our external senses. But, as similar diseased conditions commonly produce the same or similar results, we infer from the occurrence of the latter the existence of the former. It is thus that we determine as to the existence of phrenitis, pneumonia, pleuritis, laryngitis, and other maladies beyond the reach of sight and touch; it is this mode of reasoning which, being amongst the elementary principles of medical logic, constitutes the foundation of most of our pathological conclusions and practical inferences. When, therefore, in certain complaints not necessarily productive of the croup-like inspiration, we find the two co-existing, and more especially if such complaints are notoriously apt to produce or to be accompanied by enlargement of the glands of the absorbents of the neck or thorax, we shall probably rarely err if we adopt the conclusion, from the coincidence of the laryngeal affection, that there is in reality concurrent intumescence of the glands." 192.

Again:—

"But the pathological condition of these glands only establishes the liability to the attacks; the paroxysms generally require for their production the intermediate agency of some other event which is to be considered as their exciting cause, just as happens in another formidable malady, the angina pectoris, in which both the respiratory function and the action of the heart are temporarily suspended, and in which, though commonly depending upon structural disease of the coronary arteries which is a permanent morbid condition, some additional physical or moral agency is generally required to produce a paroxysm.* It is, therefore, a second, but far from secondary point in the treatment of the laryngismus stridulus, to distinguish, and to prevent or counteract, the operation of these causes of the paroxysms." 193.

---

* "In the case of an illustrious surgeon, who fell a victim to the disease, 'the first attack was produced by an affection of the mind, and every future return of any consequence arose from the same cause; and, although bodily exercise or distention of the stomach brought on slighter affections, it still required the mind to be affected to render them severe.'—*Life of John Hunter, by Sir E. Home.*"

The analogy between angina pectoris and this disease is not very good. In the first place, ossification of the coronary arteries is quite given up by all pathologists of the present day. In the next place, genuine angina pectoris, in a majority of cases, presents no appreciable organic lesion before or after death—the disease being, in fact, a neuralgia, rather than change of structure. If a neuralgic affection, therefore, is capable of producing the dreadful symptoms of angina pectoris, and even death itself—so might a nervous affection produce laryngismus stridulus.

But to return. Besides attacking the predisposing and exciting causes, and thus preventing the disease, we must resort to remedies for the purpose of checking the paroxysm itself, which often threatens death. And here our author goes over again the long list of predisposing and exciting causes, which occupied so much space under their own heads previously. Thus, age, constitution, climate, seasons, diet, dress, &c. are all *re-discussed*, in respect to their *remedial* agency, in contradistinction to the same agents in an etiological character. This plan is, no doubt, logical, but, in some respects, it is rather prolix. If, for example, we are told in one chapter, that bad diet and damp localities predispose to laryngismus stridulus, it is hardly necessary to have another chapter to inform us, that good diet and dry situations are defences against the said disease. Under the head of DIET, however, we find some excellent observations, though they certainly would have come in more appropriately under the head of causes than prevention of the disease under consideration. We shall here introduce an extract respecting the bringing up of children by hand.

"It is thus alone that, in the absence of speech, we can investigate the ailments of children; but it is a mode of physical expression which, being little under disguise, and liable neither to the exaggerations of the hypochondriac or morbidly sensitive, nor to the extenuation of those who fear the visits, the restrictions, or the doses of the doctor, is perhaps as little liable to misconception as even the articulate language of the adult. This method of inquiry has been well noticed and not unduly eulogized by Underwood and others, but particularly by the late Dr. Clarke, who says, 'It has been argued that the diseases of infant children could not be investigated, because they are not capable of describing their complaints, and that a physician, on that account, cannot understand them. This, however, is not altogether true. Their colour, figure, fatness, leanness, paleness, redness, the tone of the skin, the general or local heat or coldness, gestures, cries, brightness or dulness of the eyes, the state of the pupil, the direction of the eyes, the state of the ossification, the straitness or deformity of the skeleton, the tone or atony of the muscular fibres, the state of the abdomen, enlargement of the viscera, the state of the respiration, the various secretions, the excretions, their number, colour, consistence, texture, smell—all these, and many more symptoms of the state of health or disease present themselves to the consideration of an attentive physician, and leave him without an excuse if he neglects a part of his duty in which the feelings of parents and the good of society are so materially concerned, as in the cure of the diseases of children.'*

Amongst the functions and organs thus enumerated, the state of the alimentary canal has peculiar interest, because here will be first manifest the evidence of the injurious consequences of this mode of rearing. Whilst the evacuations

* Commentaries on the Diseases of Children, p. 34.

continue healthful, there can be no reason for alarm or interference. The appetite being unimpaired and the digestion perfect (the latter being proved by the appearance and due frequency of the stools, together with the absence of intestinal pain), the body will be well nourished, the sleep uninterrupted, the spirits cheerful, and the whole child will present a healthful aspect. But the very earliest tokens of disorder require attention. At first, probably, the food will merely pass through the canal undigested; if milk has been the principal article of diet, it will appear curdled in the evacuations; if farinaceous substances, especially arrow-root, barley, flour, or even bread have been employed, there will appear in the stools a powdery mass, pasty when recently discharged, and when dry easily broken down like baked flour, though cemented by the secretions of the canal and tinged with bile. As yet, however, there may be no disordered secretions. The colour of the evacuations may be good at the time of their expulsion, but, from exposure of the undigested food in its moistened condition to atmospheric influence, it turns sour; and the acid thus generated, as in the experiment with vinegar upon the colouring matter of bile by Tiedemann and Gmelin,* corroborated by others, tinges after a time the whole mass of a green colour.

The above is, commonly, the first in the series of injurious appearances. Afterwards, the undigested aliment brought in constant contact with the sensitive mucous lining of the canal will irritate its secreting apparatus, and produce slimy, discoloured, offensive discharges from the bowels, not unfrequently tinged or streaked with blood, combined with little if any true feculent matter, and commonly attended with much abdominal pain. Under such circumstances, alimentary materials feed disease, but *not* the constitution; the child screams from attacks of intestinal spasm; assimilation is defective; the countenance no longer preserves its roundness and healthy character, nor the limbs their plumpness; the skin, pale and flabby, hangs loosely upon the extremities; the muscles lose their firmness and contractile power; this occurring in the abdominal parietes —Nature's waistband—they offer little resistance to the distention of the intestinal canal from flatulency, and the infant has that peculiar combination, so constantly observed in ill-nourished children, of tumid and tense belly with extenuated limbs. The bones, also, losing their solidity, become twisted from posture, from pressure, or from the action of their own muscles; and, if there be any constitutional propensity to glandular enlargement, it will next to inevitably occur in those parts in which such morbid condition is particularly apt to occur, as in the neck or mesentery, and not unfrequently in both.

The removal of these undigested materials and acid contents of the bowels is necessary; and this must be effected by the mildest possible aperients, as a little castor oil, magnesia, or a little rhubarb and magnesia; or, where there is not yet any manifest disorder of the secretions, by emollient glysters, which hurry the passage through the intestinal tube of that which, if retained, would, prove a source of irritation. These will also soothe the bowels, abundantly prone under these circumstances to excitement, rather than add to the irritation which is too frequently the result of acrid cathartics; and will thus interrupt the series of evil consequences. Much may also be done by the regulation of the diet, trying the various alimentary substances usually employed for the food of children, according to the principles of selection already noticed, until some one kind shall be found to agree. If all kinds of farinaceous substances ferment, distend, or irritate, or if they even pass through undigested, it may be necessary to have recourse to animal substances, as the 'hartshorn jelly,' so much recommended by Underwood, or the 'weak mutton, chicken or beef broth, clear and free from fat, mixed with an equal measure of mucilaginous or farinaceous

---

* Recherches sur la Digestion. Prem. Partie, p. 74.

decoctions' directed by Clarke; and there may be instances in which it may be requisite to use these animal decoctions uncombined." 213.

Under the head of scrofula, Dr. Ley enumerates the long list of remedies that have been held as specifics in this *opprobrium medicorum*. He is of the same opinion with ourselves, that there is no specific for this malady, and that all the boasted remedies are useful at times, and only when properly employed. Dr. Ley dwells judiciously on the good effects of change of air —especially of change from town to country—on scrofulous children. In fact, his section on scrofula, as one of the predisposing causes of laryngismus stridulus, is a very good dissertation on the disease—a much better one than many of those monographs on scrofula, which are published *ex professo*, and with the utmost formality. Dr. Ley, indeed, will be more frequently quoted—or rather *appropriated*, by authors, for those diseases of which he does *not* treat, than for laryngismus stridulus. The reason we have before explained.

As inflamed scalp sometimes produces enlargement of the cervical glands, so soothing applications, as warm water, milk and water, or even poultices, followed by proper ointments, will be judicious.

*Difficult dentition* is by far the most frequent of those local irritations which lead to lar. stridulus, through the medium of enlarged glands;—hence the propriety of lancing the gums—and that at an early period.

"When, therefore, the crowing inspiration occurs before the deciduous teeth have reached even nearly to the surface of the gums, and the cervical glands are found, or even suspected to be, enlarged without other assignable cause, we should direct our attention to the teeth, and we shall be abundantly justified in having recourse to an '*anceps remedium potius quam nullum*.' We ought to lance the gums and divide the membrane of those teeth, which, according to their usual order of succession, may be expected to be advancing." 242.

*Cerebral congestion* may be the cause, coincidence, or consequence of the disease in question. But it is clear that, where the symptoms of head-affection exist, we ought to counteract them by the usual means. These, of course, we need not detail. The same reasoning will apply where there are evidences of pulmonary, bronchitic, or cardiac inflammation. We need not re-open the various other occasional causes, as straining of the body, immoderate exercise, fretting, cough, abdominal distention, &c.; all these must be obviated by means which are obvious to every well-informed practitioner. On these points, our author has descanted copiously and sensibly—though, perhaps, somewhat tediously. We shall, therefore, pass on to the—

### TREATMENT OF THE PAROXYSM.

At the head of the list our author places the warm-bath, in which the child should remain during the whole of the attack—at all events for a quarter of an hour. The temperature should be about 98°. Whilst in the bath, cold water should be sprinkled on the face, which is generally succeeded by a short inspiration and a lengthened expiration—and this again by a scream, which usually puts a period to the paroxysm. It is also useful to apply ammonia, if at hand, to the nostrils. Irritation of the pharynx by a feather will sometimes induce vomiting, which will of course solve the spasm of the glottis. Striking the child on the back, or roughly shaking it about, is supposed by nurses to be a good process—and it is so—but, according to our author, its utility depends on its causing the child to cry. Millar asserts that purging will solve the paroxysm—probably by throwing the muscles of the chest into action. We cannot wait for the operation of a purgative given by the mouth. An enema will be best, and into its composition should enter assafœtida, or turpentine if at hand. In such urgent and dangerous cases, our author asks, might not the forcible introduction of a tube into the glottis—or tracheotomy itself, be resorted to with advantage? We think it would be exceedingly difficult to introduce a tube into the larynx, during the convulsive efforts of a child in this state—it is not very easy in the apparently lifeless body of a person in a state of asphyxia. But tracheotomy ought, undoubtedly, to be had recourse to, if other means fail. A lancet is all that is necessary to effect the operation, as people must not be nice on such occasions. The wound can be kept dilated by a piece of curved card. The operation has been successful in the hands of Dr. Marsh, of Dublin.

"Such are the practical suggestions which I have to offer, as to the treatment of those most frequent forms of the crowing inspiration, in which the constriction of the glottis arises from injurious compression, by tumid absorbent glands, of the nerves which supply the trachea and those muscles whose office it is to open the rima glottidis, when requisite for the purpose of respiration. In the present state of our knowledge, it were too much to assert that no other cause is capable of producing this ailment by direct influence; and so general is the belief that head affection is one of those causes which may operate by direct agency, that it is little short of heresy to doubt it. The truth of this doctrine I have already seen reason to call in question; but it is also vague and unsatisfactory. It is not very distinctly stated whether it is congestion, simple irritation, inflammation or pressure of the brain or its membranes, which produces the effect, or whether the same condition, be it spasm or be it palsy, obtains through both stages of the *maladie cérébrale*, as this head-affection of children has been well denominated by Cruveilhier; and, further, the doctrine affords no satisfactory explanation of the symptoms, even if true. It does not in the least explain the fact, why the par vagum should be affected to the exclusion of other nerves, having their origin in the same respiratory column or passing close to them; nor why, if the glottis be closed by a spasm, the superior laryngeal branch of that nerve should be affected to the exclusion of the inferior and of those other branches, which proceed from the same common trunk; nor why, if the closing of the glottis be the result of paralysis from pressure at the origin of the respiratory nerves, the recurrents should be exclusively influenced; and still more difficult is it to account for the transfer of morbid influence from one branch of a common trunk to another, in the course of the same malady, which the phenomena would imply. The stage of inflammation of the brain is commonly attended with convulsive movements; that of effusion by paralysis extending even to the sphincters; yet, in both, the crowing inspiration may occur. If this arise directly, not intermediately, from the cerebral affection, the closing of the glottis must in the inflammatory stage be convulsive from excitement of the superior laryngeal nerve, whilst the recurrent, retaining only its ordinary power, is unable to counteract the muscular spasm produced by the morbid excitement of its antagonist nerve; in the stage of pressure from effusion, when all the functions and faculties are torpid from paralysis, the same closing can only arise from a palsy of the recurrent, the superior laryngeal nerve still retaining its powers unimpaired. Both can scarcely be simultaneously affected; for the equal contraction of antagonist muscles, upon which these nerves are respectively distributed, would probably keep the chink in its medium state, which is that of permeability to air; whilst paralysis of both would, in conformity with the experiment of Magendie, who annihilated all nervous influence in the four laryngeal branches of the par vagum by their division, cause the chink to be permanently open. Such transmutation of morbid influence from one nerve to another, or, what is infinitely more difficult to imagine, from one branch of a common trunk to another in the progress of the same disease, is all but inconceivable; and the improbabilities, therefore, of this opinion militate greatly against its reception. Still the opinion has maintained its ground, and has exercised great influence, nor would I denounce the treatment founded upon it as universally improper; for such a cerebral disease may be the original, though often indirect cause of the crowing inspiration, and the primary affection must be actively treated, or the patient will fall a victim to the complaint, either by general convulsions or its succeeding coma, or, sometimes, by the intermediate agency of the crowing inspiration, which is in such cases a secondary complaint. But the existence of this cerebral malady will be recognized by signs peculiarly its own, without reference to the croup-like inspiration, and the treatment will be wholly uninfluenced by its accidental combination with the laryngeal affection." 276.

This brings us to the end of the work—the Appendix, "ON THE GENERAL PATHOLOGY OF THE NERVES," being reserved for another analytical article in our next Number. We have presented as copious an analysis as any author or reader could desire—or fairly expect. We have allowed Dr. Ley to support his doctrine almost as freely as in his own book. We admit that the ingenious author has made out a strong case in favour of his theory—and, if we do not profess ourselves to be converts, it is because, as we before observed, we are unable to compel our faith or belief. Prejudice is more easily formed than eradicated. The few cases of this disease which we have witnessed, and one of which is on record, were nearly all attended by the "CARPO-PEDAL SPASM," which appellation we believe we were the first to give it. Now this association, which Dr. Ley admits as not very infrequent, shakes our confidence in the glandular pathology of the disease, and inclines us to look to other causes, and especially to IRRITATION of the gastro-intestinal nerves, the source of so many spasmodic (we hope the author will pardon the term) affections of distant parts, particularly in children, whose intestinal tube is almost the centre or focus of sensibility, irritability—we had almost said of VITALITY. A long experience and attentive observation have presented us so many examples of the strange and mysterious freaks which Nature plays in cases of gastro-intestinal irritation, both in the young and in the old, that we are not disinclined to include "LARYNGISMUS STRIDULUS" in the category. This impression is not lessened by the recollection of the many cases of strumous disease which we have witnessed, and where the cervical and other glands were developed, as it were, in proportion to the attenuation of all other parts of the body—and yet where no laryngismus stridulus was recognized.

But we will not urge any further objections, leaving it to time, and the investigation of future observers, to corroborate or refute the doctrine of our author. It would scarcely detract from the merit of the work, if the whole of Dr. Ley's hypothesis was proved to be an ingenious fiction of the imagination. The mine of facts and observations which he has laid open to his brethren, will prove an inexhaustible treasure, from which future authors will draw, with advantage, long after his and our bones shall have mouldered into dust!

